# Phage Therapy for *Acinetobacter baumannii* Infections: A Review on Advances in Classification, Applications, and Translational Roadblocks

**DOI:** 10.3390/antibiotics14111134

**Published:** 2025-11-08

**Authors:** Yilin Wang, Liuyan Li, Yuqi Liang, Kehan Xu, Ying Ye, Maozhang He

**Affiliations:** 1 The Second Clinical School of Medicine, Anhui Medical University, Hefei 230032, China; 2 Department of Microbiology, School of Basic Medical Sciences, Anhui Medical University, Hefei 230032, China; 3 The First Affiliated Hospital of Anhui Medical University, Hefei 230022, China

**Keywords:** CRAB infection, antibiotic resistance, bacteriophage classification, phage cocktail therapies, genetic engineering

## Abstract

The global spread of carbapenem-resistant *Acinetobacter baumannii* (CRAB) poses a severe public health threat, driving growing interest in phage-based precision antibacterial strategies. This systematic review synthesizes recent advances in the field of *A. baumannii* phage. Modern taxonomy, based on whole-genome phylogeny, has reclassified the majority of *A. baumannii* phages into the class Caudoviricetes, revealing distinct evolutionary clades that correlate with host tropism and biological properties, superseding the traditional morphological families (Myoviridae, Siphoviridae, Podoviridae). To overcome limitations of natural phage therapy, such as narrow host range, cocktail therapies (ex vivo resistance mutation rates < 5%) and phage-antibiotic synergism (enabling antibiotic efficacy at 1/4 minimum inhibitory concentration) have significantly enhanced antibacterial efficacy. Preclinical models demonstrate that phage therapy efficiently clears pathogens in pneumonia models and promotes the healing of burn wounds and diabetic ulcers via immunomodulatory mechanisms. Technical optimizations include nebulized inhalation delivery achieving 42% alveolar deposition, and thermosensitive hydrogels enabling sustained release over 72 h. Genetic engineering approaches, such as host range expansion through tail fiber recombination and CRISPR/Cas-mediated elimination of lysogeny, show promise. However, the genetic stability of engineered phages requires further validation. Current challenges remain, including limited host spectrum, the absence of clinical translation standards, and lagging regulatory frameworks. Future efforts must integrate metagenomic mining and synthetic biology strategies to establish a precision medicine framework encompassing resistance monitoring and personalized phage formulation, offering innovative solutions against CRAB infections.

## 1. Introduction

*Acinetobacter baumannii* is a Gram-negative opportunistic pathogen renowned for its remarkable environmental persistence—it can survive for extended periods on dry surfaces and exhibits significant resistance to conventional disinfectants [1,2,3]. This bacterium is a core pathogen in hospital-acquired infections (particularly in intensive care units), commonly causing ventilator-associated pneumonia (VAP), bloodstream infections, and wound infections, posing a life-threatening risk to immunocompromised patients, those with severe burns/trauma, and those with prolonged mechanical ventilation [4,5,6]. Its pathogenicity depends on multiple virulence factors: outer membrane protein A (OmpA) mediates host cell adhesion and immune evasion; porins regulate substance exchange; enhanced biofilm formation promotes environmental adaptability; an efficient iron uptake system promotes survival within the host [7]; The capsule effectively resists phagocytosis by host phagocytes, serving as a crucial physical barrier against host immune clearance; lipopolysaccharide (LPS), particularly its lipid A component, is a key pathogen-associated molecular pattern that activates the host innate immune response [8]; the secretion system can deliver effector proteins to host cells or competing bacterial species [9]. As a key member of the “ESKAPE” group of drug-resistant pathogens, its widespread multidrug resistance (MDR) has been classified by the World Health Organization (WHO) as a Critical Priority threat [10].

Phages, as bacteria-specific lytic agents, offer an alternative strategy for addressing drug-resistant bacterial infections [11]. Phages exhibit numerous significant advantages in the treatment of *A*. *baumannii* infections. First, phages exhibit precise targeting: their infection begins with the high-affinity binding of tail filament proteins to specific receptors on the surface of *Acinetobacter baumannii* (such as capsular polysaccharides, lipopolysaccharide, or outer membrane proteins). Through receptor-ligand interactions, they specifically recognize host strains, maximally protecting the commensal microbiota and reducing the risk of microecological disruption [12,13]. Additionally, bacteria develop resistance to phages at a slower rate than they do to antibiotics [14]. This is because evading phage attachment often requires sacrificing or altering key surface structures (such as the capsule), which may weaken bacterial virulence or increase sensitivity to antibiotics [15]. This opens new avenues for addressing antibiotic resistance. Furthermore, phages achieve self-amplification at the infection site through the lytic cycle, continuously increasing local effect concentrations and enhancing pathogen clearance [16].

Currently, the theoretical basis and practical achievements of phage therapy have been gradually validated. The theoretical basis for phage therapy in treating *A*. *baumannii* infections primarily includes phage-host interaction mechanisms and cocktail therapy design. Phages highly depend on their tail structures, particularly tail fibrins, to specifically recognize bacterial surface receptors (such as OmpA, lipopolysaccharide (LPS), capsular polysaccharide (CPS), etc.) [17]. The genetic diversity of tail fibrins determines the breadth of the host spectrum [18]. Cocktail therapy design expands strain coverage and delays the emergence of resistance by combining phage strains with complementary host spectra [19]. Empirical studies have shown that in animal models (mouse/rat pneumonia), phages can effectively eliminate carbapenem-resistant strains, significantly improve survival rates, and alleviate pulmonary pathological injury [20,21,22]; At the clinical level, personalized phage cocktail therapy shows potential in the treatment of severe infections, and early randomized controlled trials (RCTs) provide preliminary evidence for its translation [23,24,25].

In the face of the global epidemic crisis of multidrug-resistant *A*. *baumannii*, phage therapy, with its unique mechanism of action and growing body of evidence, emerges as a highly promising solution [26]. This review will: (1) systematically analyze the molecular mechanisms underlying phage-*A. baumannii* interactions; (2) summarize advances in phage taxonomy based on genomics; and (3) review key findings from preclinical studies and clinical translation, aiming to provide mechanistic insights and translational pathway design support for advancing precision phage therapy in clinical practice.

## 2. Current Drug Options for the Treatment of *A. baumannii* in Clinical Practice

### 2.1. Drug Resistance Mechanisms of Acinetobacter baumannii

The resistance mechanisms of *Acinetobacter baumannii* are complex and diverse, involving multiple molecular pathways and specific resistance strategies against various drug classes. Among these, the overexpression of antibiotic efflux pump genes [27] represents a key mechanism driving antibiotic resistance. The efflux pump systems of *A. baumannii* expel harmful substances such as antibiotics out of the cell, thereby reducing intracellular drug concentrations and conferring resistance to bacteria. Currently known efflux pump systems in *A. baumannii* primarily include the RND family, MATE family, and MFS family. Their mechanisms may be influenced by multiple factors: on one hand, bacteria may increase efflux pump gene expression levels through mechanisms such as genetic mutations; on the other hand, environmental factors may also affect efflux pump expression. For example, the presence of antibiotics may induce bacterial efflux pump gene expression to adapt to drug pressure [27]. Compared to wild-type strains, pore protein mutations (e.g., OprD) result in unique pore diameters and reduced anion selectivity, while pore protein deletions lead to decreased membrane permeability. These alterations reduce the entry of antibiotics and other drugs into the cell, thereby enhancing bacterial resistance [28]. Overexpression of efflux pumps in *A*. *baumannii* often synergizes with pore protein deletion to form a “double barrier” effect, substantially reducing intracellular drug concentrations. *A. baumannii* can hydrolyze carbapenem antibiotics through the production of multiple β-lactamases (e.g., OXA-type carbapenemases, AmpC cephalosporinases), rendering them inactive. OXA-type β-lactamases represent one of the most prevalent mechanisms for carbapenem resistance in *A. baumannii* [29]. Topoisomerase mutations also confer high-level multidrug resistance. Multiple studies have demonstrated the emergence of gyrA and parC gene mutations during *A. baumannii* hospital outbreaks. Among these mutations, specific amino acid codon substitutions were identified, such as those occurring in the gyrA gene and within the quinolone resistance determinant region of the parC gene. These mutations are strongly associated with *A*. *baumannii* resistance to fluoroquinolone antibiotics [30]. Biofilms are structures formed when bacteria are enveloped by a polymeric matrix secreted by the bacteria themselves. The extracellular matrix of biofilms can delay the entry of antimicrobial agents into the biofilm interior and hinder their diffusion. Research indicates that antibiotic resistance is closely associated with the formation of *A*. *baumannii* biofilms [31], but the specific mechanisms underlying biofilm formation in *A. baumannii* remain incompletely understood. Additionally, mechanisms such as ribosomal protection, lipopolysaccharide modification, and heterogeneous resistance also contribute to the emergence of multidrug-resistant and even pan-resistant strains.

### 2.2. Treatment of CRAB

According to the 2024 guidelines of the Infectious Diseases Society of America (IDSA), sulbactam-durlobactam combined with a carbapenem is the first-line treatment for CRAB pulmonary infections [32,33]. Durlobactam, as a novel β-lactamase inhibitor, effectively inhibits OXA-type carbapenemases, restoring sulbactam’s antimicrobial activity against *A*. *baumannii* [34,35]. Clinical studies have shown that it significantly reduces mortality rates in CRAB pneumonia. For strains sensitive or intermediate to sulbactam (MIC ≤ 8 μg/mL), high-dose ampicillin/sulbactam (sulbactam 6–9 g daily) can be used as an alternative regimen, but it must be combined with other antimicrobial agents (such as polymyxin, minocycline) to enhance efficacy [36]. Note that this regimen is ineffective against sulbactam-resistant strains (MIC ≥ 16 μg/mL). Polymyxins (including polymyxin E [colistin] and polymyxin B) are traditional drugs for treating CRAB infections [37]. The IDSA guidelines recommend prioritizing polymyxin B due to its relatively lower risk of nephrotoxicity and more stable pharmacokinetic properties (the AUC/MIC ratio associated with dose-dependent nephrotoxicity is more controllable) [32]. In contrast, colistin therapy often fails to achieve adequate exposure due to a narrow therapeutic window and insufficient lung tissue penetration, and monotherapy may increase the risk of treatment failure and resistance [38]. Among tetracycline derivatives, tigecycline and minocycline exhibit some activity against CRAB [39]. Tigecycline can circumvent classic tetracycline resistance mechanisms such as tet(M) (by blocking the action of ribosomal protection proteins through steric hindrance rather than “higher affinity”) [40], but its standard dose (50 mg q12h) is insufficient in lung tissue and has low blood concentrations, so it is not recommended for monotherapy in bacteremia. Clinically, high-dose regimens and combinations with other antibacterial drugs are required [41,42,43]. Minocycline, a semi-synthetic tetracycline, is often used in combination with carbapenems, polymyxins, or rifampin, showing synergistic effects against some CRAB strains [44]. Its pharmacokinetic properties are superior to those of tigecycline, and high-dose administration (200 mg q12h) is recommended, but the optimal dose still needs to be supported by evidence-based research [45]. Cefiderocol is an iron-tethered cephalosporin. In its phase III clinical trial (CREDIBLE-CR), the all-cause mortality rate in the CRAB infection subgroup was numerically higher than that in the control group (the specific reason is unclear) [46]. Based on this, both the ESCMID and IDSA guidelines restrict its use: it should only be considered for refractory infections where other treatment options have failed, and caution should be exercised regarding potential heterogeneous resistance during treatment [32,47].

### 2.3. Treatment of Carbapenem-Susceptible A. baumannii

For non-multidrug-resistant (non-MDR) *A*. *baumannii* infections, clinicians must balance the severity of the infection with antimicrobial stewardship requirements [45]. Amoxicillin/clavulanate and carbapenems (imipenem, meropenem) have comparable efficacy against susceptible strains and can be considered as first-line options for mild to moderate infections [48]. Fourth-generation cephalosporins (e.g., cefepime) should only be considered when susceptibility testing confirms sensitivity, as clinical efficacy data are limited [49].

The resistance in *A*. *baumannii* is alarming: global surveillance shows that carbapenem resistance rates (CRAB) have exceeded 80% in some regions, and there is a high rate of cross-resistance to aminoglycosides and tetracyclines [50]. More seriously, the resistance rates of polymyxin and tigecycline, the traditional “last line of defense” drugs, continue to climb. At the same time, the research and development of new antibiotics has lagged far behind: in the past decade, only a few new drugs such as cefiderocol and sulbactam/dorlobactam have been approved, and their applicable populations are limited [50,51,52]. Antibiotic resistance not only directly increases patient mortality but also significantly prolongs hospital stays, resulting in a substantial healthcare burden [53]. In the face of the threat posed by extensively drug-resistant (XDR) or even pan-drug-resistant (PDR) strains, the development of non-antibiotic novel therapies has become an urgent priority.

Confronted with the escalating challenge of antimicrobial resistance and the constrained pipeline of conventional antibiotics, the field is compelled to explore innovative paradigms. Bacteriophage therapy represents one such compelling avenue, whose rational development and application require a foundational knowledge of phage biology, which we elaborate on next.

## 3. Morphological, Genomics and Taxonomy of *A. baumannii* Phages

The head of a bacteriophage typically has an icosahedral structure containing a single type of genetic material (DNA or RNA) tightly enclosed by proteins. The head is connected to the tail apparatus via a neck ring structure, with the tail core components including a retractable tail sheath and hollow tail tube. During infection, the contraction of the tail sheath drives the tail tube to penetrate the host cell membrane, enabling the genome to be injected into the cytoplasm via the tail tube lumen. This mechanism ensures the stability and efficiency of nucleic acid transfer [54]. The tail structure (especially the tail fibers) serves as a key molecular machine that mediates host-specific recognition and genome delivery [17]. The tail fiber proteins of bacteriophages are composed of receptor-binding domains (RBDs), whose amino acid sequences exhibit high genetic diversity. Different RBD variants can specifically recognize receptors on the surface of *A*. *baumannii* (such as OmpA and LPS), thereby determining the breadth of the phage’s host range. Based on the targeting specificity of RBD, the genes encoding it can serve as molecular markers for the development of detection tools for *A*. *baumannii* [18].

According to the latest taxonomy from the International Committee on Taxonomy of Viruses (ICTV, 2025), the classification of *Acinetobacter* phages has undergone a fundamental shift from a morphology-based system to one rooted in whole-genome sequencing and phylogenetic analysis [55]. The traditional order Caudovirales and its constituent families—Myoviridae, Siphoviridae, and Podoviridae—have been abolished. These groups were found to be polyphyletic, meaning they did not accurately represent evolutionary relationships. The vast majority of known *A. baumannii* phages are now classified within the class Caudoviricetes (tailed phages). Under this new framework, phages are assigned to various orders and families based on genomic similarity and core gene phylogenies, which more reliably reflect their true evolutionary history.

While morphological terms like “myovirus-like” (contractile tail), “siphovirus-like” (long, non-contractile tail), and “podovirus-like” (short tail) remain useful for descriptive purposes, they are no longer formal taxonomic ranks. Research on non-tailed phages (e.g., Leviviricetes, Faserviricetes) infecting *A. baumannii* remains limited, and their host recognition mechanisms and ecological roles require further investigation.

### 3.1. Overview of Modern Genomics-Based Taxonomy

Under the latest taxonomy, phages infecting *A. baumannii* primarily belong to the class Caudoviricetes, and their genetic diversity is reflected in a variety of different genera and families.

#### 3.1.1. Myovirus-like Phages

**Figure 1:**
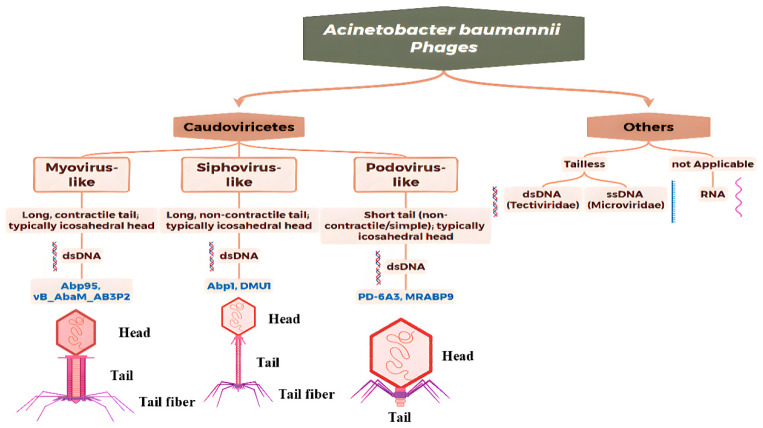
Classification based on nucleic acid types of *Acinetobacter baumannii* phages.

Based on the isolated *A. baumannii* phage, phages in this morphological group share the common morphological feature of a contractile tail, whose tail sheath is composed of multiple protein subunits arranged in a helical pattern along the tail tube, which plays a crucial role in the phage infection process (Figure 1, Table 1). Phages in this group often exhibit significant potential for therapeutic applications because of their strong lytic activity and large genomic capacity (typically >100 kb), making them promising biological agents for addressing multidrug-resistant *A. baumannii* infections. Their high lytic activity can rapidly eliminate resistant bacteria, whereas the auxiliary metabolic genes (such as DNA-modifying enzymes) carried in their genomes may enhance bactericidal efficiency by regulating host metabolism [56].

Phagecoctavirus: vB_AbaM-DLP_1/2 features a long ellipsoidal head and contractile tail, with high bursting capacity, short latency period (approximately 20 min), and good stability within a certain pH range, meeting the criteria for treatment candidates against *A. baumannii* [57].

Obolenskvirus: Phage Abp95 has a relatively limited host range, but its short latency period, high burst size, rapid adsorption characteristics, and the encoded capsular depolymerase make it a potential therapeutic candidate targeting CRAB [58]. Genomic analyses indicate that phages vB-AbaM-IME-AB2, WCHABP1, WCHABP12, BUCT628 [59], and HZY2308 [60] also belong to the genus Obolenskvirus.

Unclassified Phages: Compared with similar phages, vB_AbaSi_W9 demonstrated a broader host range, and despite its lower lytic efficiency, it is still considered a potential therapeutic candidate for CRAB infection [61]. vB_AbaM_AB3P2 (head diameter 70 nm, tail length 100 ± 10 nm) exhibits strong lytic activity but specifically lyses only AB3 and AB9 strains, with a narrow host range [62]. Additionally, a novel temperate phage, vB_AbaM_ABMM1, demonstrated rapid adsorption, strong lytic activity, a high burst plaque formation, and effective antibacterial efficacy both in vitro and in vivo. Nevertheless, concerns persist about the therapeutic risks related to lysogenic conversion [63]. Phab24 [64], vB-GEC_Ab-M-G7 [65] and vB_AbaSi_W16 [66] have been reported to exhibit morphological and genomic characteristics similar to those of the Myovirus-like Phages.

#### 3.1.2. Siphovirus-like Phages

Phages in this morphological group possess long, non-contractile tails. Morphological features such as tail length, diameter, and the number and arrangement of tail fibers often vary significantly among different phages, contributing to their structural diversity. *A. baumannii* phages belonging to this group typically exhibit a narrow host range [63].

Friunavirus: Phage Abp1 possesses a typical icosahedral head and a prominent, slender, noncontractile tail. Its genome is approximately 40–50 Kilobase (kb) in size and encodes multiple proteins associated with host adsorption and biofilm penetration. It exhibits high specificity toward the *A. baumannii* AB1 strain [67].

Unclassified Phages: Phage DMU1 possesses a flexible long tail structure with transverse striations, and the tail tip potentially harbors tail spikes and/or tail filaments. However, its host range is narrow, infecting only standard *A. baumannii* strains ATCC 19606 and ATCC 17978 [68]. Recently, Rastegar et al. reported the antimicrobial potential of vB_AbaS_SA1 against multidrug-resistant clinical isolates of *A. baumannii*. This bacteriophage exhibited a 20 min incubation period and a burst size of approximately 250 PFU/cell, demonstrating its potential therapeutic application value [69].

#### 3.1.3. Podovirus-like Phages

Phages in this group have short, non-retractable tail structures.

Friunavirus: Studies have shown that bacteriophage PD-6A3 maintains high activity within a temperature range of 4–50 °C and a pH range of 5 to 10, and can complete over 90% of host adsorption within 5 min [70]. Phage MRABP9 exhibits a short latency period, high burst yield, significant antibiofilm activity, and strong bacterial regeneration inhibition capacity, while also demonstrating excellent environmental stability [71].

Unclassified Phages: vB_AbaSi_W8 exhibited lytic activity against clinical CRAB strains. Spot tests showed that they lysed 24.14% and 34.48% of the tested strains, respectively, and formed clear lytic plaques. However, the host range of vB_AbaSi_W8 was narrower than that of vB_AbaSi_W9 [61]. The newly discovered bacteriophage vB_AbaAut_ChT04 has an incubation period of approximately 10 min, a burst size of 280 PFU/cell, and can infect 52 clinical MDR-AB strains (out of a total of 150 test strains), demonstrating a broad host range and promising application prospects [72].

### 3.2. Development of Taxonomic Basis and Crucial Genomic Characteristics

The classification of bacteriophages has undergone a fundamental shift from morphology-based systems to a genome-based phylogenetic framework. This transition has resolved significant inconsistencies between phenotypic appearance and genetic relatedness, revealing that traditional tailed morphology families (Myoviridae, Siphoviridae, Podoviridae) are polyphyletic. The current taxonomy, as ratified by the International Committee on Taxonomy of Viruses (ICTV), abolishes these morphology-based families and classifies all tailed phages within the class Caudoviricetes, with the primary basis for classification being genomic similarity and phylogenetic analysis of core genes [55,73].

This paradigm shift is powerfully illustrated by the genus Friunavirus. While historically associated with the Myoviridae family due to their contractile tails, genomic analyses now place Friunavirus within the Caudoviricetes. More importantly, this genus exemplifies the limitation of tail morphology as a definitive taxonomic marker. Comparative genomics has revealed that some phages with Siphoviridae-like (long non-contractile tail) or Podoviridae-like (short tail) morphologies can share high genomic similarity and core gene phylogeny with Myoviridae-like Friunavirus members, indicating a common evolutionary origin despite morphological divergence [74]. Such discrepancies arise because morphological traits can be influenced by convergent evolution under similar host selection pressures, while the genomic core reveals the true phylogenetic signal.

The vast majority of phage genetic diversity, however, remains largely unexplored. Metagenomic studies suggest that a significant proportion of phages in the environment are classified as “microbial dark matter” because they are difficult to cultivate and thus remain unidentified [75]. Emerging techniques are now beginning to characterize this dark matter. For instance, single-cell viral tagging (VT), a technique that identifies interacting phage-host pairs by fluorescently staining viruses and sorting host cells, has revealed hundreds of rare and highly diverse phage-host pairs in the human gut that are undetectable by standard virome sequencing [76]. This confirms that the known genomic features of *A. baumannii* phages represent only a fraction of their true genetic and functional potential.

Beyond taxonomy, phage genomes encode the crucial determinants of their biology. The genetic diversity of receptor-binding domains (RBDs) in tail fiber genes is a primary determinant of host specificity [75]. Furthermore, phage genomes can carry auxiliary metabolic genes that directly enhance infectivity. A prominent example is the capsular depolymerase, which degrades the host’s protective capsule, a critical barrier for infecting encapsulated pathogens like CRAB [77]. Critically, the infection strategy—lytic vs. lysogenic—is also genetically hardwired. Temperate phages can pose therapeutic risks, as exemplified by the *A. baumannii* temperate phage Φ19606, which was found to act as a mobile vehicle for the colistin resistance gene eptA1, promoting the emergence of resistant mutants [78]. Recent research also highlights that the human host actively influences viral behavior; compounds produced by human gut cells can induce the activation of dormant temperate phages, suggesting a direct role of the host physiology in shaping the gut virome [79].

In conclusion, modern phage taxonomy is deeply integrated with functional genomics. Moving beyond morphology to decipher the genomic code—including genes for host range, depolymerases, and life cycle regulation—is crucial for scientifically selecting therapeutic candidates, assessing their safety, and harnessing their full potential to combat multidrug-resistant infections.

### 3.3. Challenges and Trends

Currently, the classification and research of *A*. *baumannii* phages face multiple challenges. A comprehensive review of *A. baumannii* phage research over the past decade highlights that these challenges—including lysogenic conversion risks, uncultured “dark matter” phages, and bacterial anti-phage defenses—are interconnected and have long constrained translational progress [80]. Lytic phages are widely present in clinical strains (accounting for over 40%) [81], posing potential therapeutic and safety risks. For example, phages belonging to the Friunavirus genus frequently carry virulence-related genes and may also form phage-plasmid chimeras, leading to the unintended transition of lytic phages—originally intended for therapeutic use—into the lysogenic cycle during infection, thereby enhancing the host bacterium’s antibiotic resistance phenotype [82]. Recent studies have further revealed that these integrated prophages themselves encode a rich array of defense systems (such as anti-phage proteins and CRISPR-like mechanisms) to resist invasion by other exogenous phages. This significantly increases the complexity of the host’s intrinsic immune barriers that must be overcome during phage therapy [83]. Although structural biology research has made significant progress in understanding host-range restriction mechanisms, recent findings indicate that CRISPR-Cas systems can establish an anti-phage barrier by cutting phage DNA, whereas Myoviridae phages can encode diverse anti-CRISPR proteins to evade this immune defense [84]. More critically, metagenomic studies indicate that a myriad fraction of phages in the environment are classified as microbial “dark matter” because of their difficulty in cultivation, remaining unidentified, and unexploited for extended periods [85]. However, emerging technologies, such as single-cell sorting and deep learning, are transforming this landscape. Adam Sidi Mabrouk and colleagues used high-throughput microfluidic devices to conduct long-term analysis and separation of phages and their effects on host cells, which helps to screen and characterize host-phage interactions more efficiently and provides new technical means and research ideas for capturing uncultivable phages [86]. Consistent with this technical direction, a recent review proposes that integrating metagenomic mining (to capture “dark matter” phages) and synthetic biology (to engineer phage defenses) will be the core strategy to address current bottlenecks, aligning with the long-term goal of establishing a precision phage therapy framework [80].

### 3.4. Bacteriophage Resistance Mechanism of A. baumannii

Beyond the morphological, genomic, and taxonomic characteristics of *Acinetobacter baumannii* phages, deciphering the resistance mechanisms evolved by bacteria under phage attack is equally crucial for illuminating their coevolutionary dynamics and optimizing phage therapy strategies [80].

Surface Receptor Alteration and Adsorption Inhibition: This represents the most common and direct strategy for *Acinetobacter baumannii* to develop phage resistance. Many phages utilize bacterial capsular polysaccharides as initial adsorption receptors. To evade infection, bacteria halt production or alter the composition of their capsular polysaccharides through genetic mutations. While this modification effectively blocks phage attachment, bacteria often incur adaptive costs. For instance, loss of the capsule frequently accompanies reduced biofilm formation capacity, diminished virulence, and increased susceptibility to complement-mediated killing and specific antibiotics [87].

Genetic Horizontal Transfer and Mobile Genetic Elements: The genome of *Acinetobacter baumannii* exhibits high plasticity, enabling it to acquire resistance elements through horizontal gene transfer. Notably, certain temperate phages themselves can serve as vectors for spreading resistance genes. For example, phage Φ19606 has been identified as capable of integrating into the host genome and acting as a mobile carrier for the polymyxin resistance gene eptA1. Simultaneously, its lysogenic cycle may also influence the host’s physiological state [78].

Intrinsic immunity and bacterial defense systems: A recent study identified a key regulatory protein named DdaA in the Moraxellaceae family (including *Acinetobacter baumannii*). Under stress conditions such as DNA damage, DdaA globally activates the expression of a series of anti-phage defense genes (including CRISPR-Cas and restriction-modification systems), recognizes and cleaves invading phage DNA, thereby providing specific immunity [88].

## 4. Application of Phages in the Treatment of *A. baumannii* Infections

### 4.1. Advances in In Vitro Research

The host range of a single phage is typically highly specific at the subspecies level. While this offers the advantage of precisely targeting pathogens without disrupting the host microbiota, it also makes it challenging to identify suitable phages [89]. Additionally, bacteria can develop phage resistance through mechanisms such as receptor mutations, exemplified by that 5 out of 23 *A*. *baumannii* strains (21.7%) exhibited resistance to a single phage, manifested by the presence of viable colonies within the lysis zone [90]. Phage cocktail therapy significantly reduces the incidence of resistance—even if bacteria are resistant to one component, they remain sensitive to other phages. This strategy achieved a coverage rate of 70% for the tested strains (7 out of 10 strains were lysed), demonstrating broader antimicrobial potential [91]. In phage cocktail therapy, there is also an optimization strategy based on complementary lysis spectra. In an in vitro study, AB-Army1 targeted the capsular factor, lysing 98% of capsular-positive bacteria post-infection; the remaining bacteria became resistant to this phage due to capsular deficiency (manifesting as transparent colonies). Meanwhile, AB-Navy1-4 specifically infected non-capsular bacteria, forming a complementary relationship with AB-Army1. Therefore, the “AB cocktail” composed of five phages can completely inhibit bacterial growth, achieving synergistic bactericidal effects [92].

When the combined effect of two drugs exceeds the sum of their individual effects, therapeutic synergy occurs [93]. In response to the emergence of phage resistance [94], phage-antibiotic combination therapy (phage-antibiotic synergy, PAS) has gained attention in recent years. Multiple in vitro studies have shown that PAS significantly improves antimicrobial efficacy and holds significant clinical potential [95]. Phage P21 completely inhibits the growth of wild-type *A*. *baumannii* WHG40004 in the presence of meropenem and imipenem [96]. When phage vB_AbaSi_W9 is used in combination with tigecycline and rifampicin, it shows significant synergistic effects on clinical strains [61]. vB_AbaS_SA1 also shows significant phage-antibiotic synergistic effects and reduces the overall effective concentration of antibiotics in time-kill assessments [69].

The synergistic mechanism primarily involves two aspects: First, the phage targets virulence factors to drive bacterial evolution into a low-virulence, phage-resistant but antibiotic-sensitive type, thereby being eliminated by antibiotics [97]. Recent in vitro studies further validate this mechanism: Bacteriophage Indie was shown to specifically resensitize multidrug-resistant *A. baumannii* strains to multiple antibiotics (e.g., meropenem, tigecycline) by downregulating bacterial efflux pump genes and restoring membrane permeability, providing direct evidence for the “phage-induced antibiotic sensitivity” pathway [98]; Second, antibiotics may disrupt the structure of bacterial defense mechanisms, promoting phage adsorption, invasion, and proliferation [99]. The synergistic effect of phages and antibiotics offers a new strategy for treating *A*. *baumannii* infections, not only enhancing bactericidal efficiency and reducing antibiotic usage but also delaying the development of resistance and lowering the risk of phage resistance [61,99]. Although in vitro results are encouraging, further research is needed to determine the optimal ratio, timing, and molecular mechanisms of phage-antibiotic combinations, and to validate their safety and efficacy through animal and clinical trials.

### 4.2. Advances in In Vivo and Clinical Research

#### 4.2.1. Respiratory System

**Figure 2:**
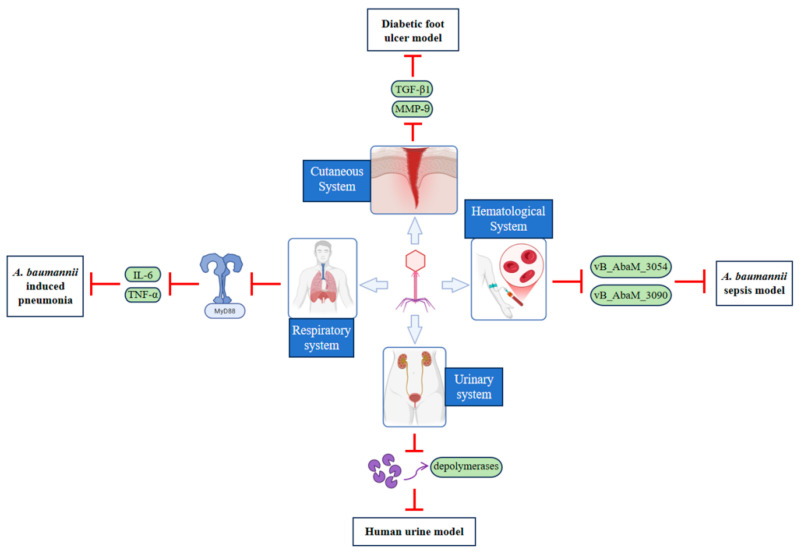
In Vivo and clinical research advances in the treatment of *Acinetobacter baumannii* infections using bacteriophage.

Multiple studies have demonstrated the antibacterial therapeutic effects of phages against *A*. *baumannii* infections in mouse pneumonia models [20,21]. However, the use of phage therapy faces significant challenges, including a narrow host range [100], the potential to induce resistance, and substantial variability in immune responses among individuals. In a rat pneumonia model, the phage cocktail significantly reduced the bacterial load in the lungs of CRAB-infected rats, achieving effective clearance of CRAB. By modulating the TLR4/MyD88 pathway, it suppressed inflammatory mediators such as TNF-α and IL-6, mitigated lung tissue damage, and prevented immune failure. (Figure 2, Table 2) [22]. In recent years, phage-antibiotic combinations have also been successfully used in clinical settings to treat ventilator-associated pneumonia [101], CRAB-induced lung abscesses, and pneumonia [102]. Nannan Wu et al. (2021) also reported the potential emergency value of phage therapy for CRAB infections in COVID-19 patients [103].

Collectively, these basic and clinical findings indicate that phage therapy, particularly the cocktail strategy, represents a promising novel approach for treating multidrug-resistant (MDR) and extensively drug-resistant (XDR) *A. baumannii* pulmonary infections. A recent study provides profound insights into the mechanisms by which phages modulate host immune responses, offering a scientific foundation for understanding their clinical efficacy and refining therapeutic protocols. Nevertheless, numerous challenges in this field warrant further investigation. Future research should prioritize the standardization of phage preparations, along with conducting larger, well-designed clinical trials to validate their safety and efficacy. In-depth exploration of the complex interactions among phages, the host, pathogens, and antibiotics is also essential. As these challenges are gradually addressed and the underlying mechanisms further elucidated, phage therapy is anticipated to evolve from a salvage treatment into a key precision therapeutic option for drug-resistant *A. baumannii* lung infections, particularly in critically ill patients.

#### 4.2.2. Cutaneous System

Due to *A*. *baumannii*’s long-term survival capability in hospital environments, coupled with its robust inherent and acquired antibiotic resistance, some strains of *A*. *baumannii* have become important nosocomial pathogens, particularly in burn units and intensive care units where they frequently cause wound infections [106]. Microbial colonization and infection of burn wounds can lead to catastrophic consequences and are one of the primary causes of patient mortality [118]. In recent years, phage cocktails have been used to treat infections caused by *A*. *baumannii*, *Pseudomonas aeruginosa*, and *Staphylococcus aureus* in burn wound patients [104]. Before using phages, it is essential to identify the infecting strain, as phages exhibit high strain specificity. Phage therapy is recommended in combination with traditional local burn wound care products, and systemic antibiotics may be administered as needed [106]. Additionally, the synergistic effect of phages with low-concentration chlorhexidine (0.02%) disrupts the three-dimensional structure of biofilms: phage-encoded depolymerase degrades the extracellular polysaccharide matrix, increasing the penetration depth of the antimicrobial agent by 2.8 times and significantly delaying the development of bacterial adaptive resistance [107].

Diabetic foot ulcers are one of the severe complications of diabetes, characterized by non-healing wounds [119]. Shivaswamy et al. found that phages exhibit wound healing-promoting effects in diabetic foot ulcer models, with mechanisms related to reduced local MMP-9 protease activity and increased TGF-β1 expression [105]. Although clinical trials have confirmed their safety, there are currently few reported cases of phage therapy for diabetic wounds, and there is a lack of double-blind clinical efficacy trials [108]. Future research urgently needs to advance rapid diagnostic technologies to guide precise phage selection, optimize local delivery systems (such as phage dressings), and design and implement rigorous randomized controlled trials, particularly in areas with urgent needs such as diabetic wounds, to advance phage therapy from case-based treatment to standardized clinical application.

#### 4.2.3. Hematological System

*A. baumannii* is a major pathogen responsible for severe bloodstream infections, including bacteremia, sepsis, and septicemia. A study by Lika Leshkasheli et al. demonstrated that the lytic phages vB_AbaM_3054 and vB_AbaM_3090—whether used individually or in combination—exhibit high efficacy in a mouse model of multidrug-resistant *A. baumannii* sepsis. These phages mediate a “targeted recognition–genome injection–bacterial lysis” cycle, enabling rapid clearance of pathogens from the bloodstream. Concurrently, they activate the complement system and enhance macrophage phagocytosis, leading to a marked increase in the 72-h survival rate of septic mice from 20% to 80%. The strict lytic life cycle of these phages and the absence of harmful genes further underscore their therapeutic safety and efficacy. These findings provide strong support for the development of phage-based therapies against drug-resistant bacterial infections [109]. However, relevant clinical reports are currently lacking.

However, the complex interactions between phages entering the bloodstream via intravenous injection and the host immune system warrant attention. Studies indicate that repeated phage injections activate non-specific immunity in animals, leading to elevated levels of inflammatory mediators such as IL-6 and TNF-α, and causing a rapid 2–3-fold decrease in phage titers within plasma within 5 min. Furthermore, the body can generate specific neutralizing antibodies, which have been detected in repeated-dose rat and crab-eating macaque models. These antibodies neutralize the phage and may contribute to treatment failure. Although existing animal models show no apparent toxicity, the accelerated phage clearance induced by immune responses and the potential reduction in therapeutic efficacy remain significant challenges for clinical translation [110].

#### 4.2.4. Urinary System

Annually, approximately 150 million people worldwide are affected by urinary tract infections (UTIs) [120]. Multidrug-resistant *A*. *baumannii* is among the key pathogens implicated in these infections [121]. The increasing prevalence of antimicrobial resistance has posed significant therapeutic challenges, leading to difficult treatment outcomes, high recurrence rates of UTIs, and substantial economic burdens [115]. In animal model studies, phages offer advantages such as not disrupting the normal microbiota, targeting resistant bacteria and biofilms, and being cost-effective [113,114]. Treatment can be administered using natural phage cocktails, lysozymes, or genetically engineered phages [111]. In vitro experiments have shown that phages also have a certain effect on *A*. *baumannii* biofilms. Grygorcewicz et al. demonstrated the efficacy of a phage cocktail combined with antibiotics against *A*. *baumannii* biofilms in a human urine model. Phage-encoded depolymerases degraded the polysaccharide matrix of the biofilm, thereby exposing the bacterial cells. Progeny phages released during the lytic cycle penetrated deeper layers of the biofilm, enabling continuous infection and disruption of the bacterial community. In the urinary environment, the combination of phages with certain antibiotics enhanced antibacterial efficacy through phage-antibiotic synergy (PAS). The underlying mechanisms included improved antibiotic penetration facilitated by phages, antibiotic-induced promotion of phage replication, and reduced emergence of resistance. Among the combinations tested, the most effective was the phage cocktail paired with trimethoprim/sulfamethoxazole [112].

#### 4.2.5. Preventing Hospital-Acquired Transmission

CRAB is a major nosocomial pathogen worldwide, with its prevalence increasingly reported in ICUs [116]. Conventional infection control strategies—including strict hand hygiene, rational antimicrobial use, contact precautions, and environmental disinfection—exhibit limited efficacy in curbing the spread of CRAB [122,123]. There is a growing need to develop novel approaches to environmental disinfection to mitigate its transmission. A study by Yu-Huai Ho et al. (2016) demonstrated that spraying an aerosolized bacteriophage formulation in ICU wards significantly reduced the colonization rate of CRAB on environmental surfaces and subsequent infection rates within the ICU, but further assessment of resistance risks and optimization of disinfection strategies are needed [117].

#### 4.2.6. Application in the Detection of Antimicrobial-Resistant *A. baumannii*

In the detection of antimicrobial-resistant *A*. *baumannii*, traditional culture-based testing methods are cumbersome and time-consuming [124], while molecular biology methods, immunological methods [125], and biosensor methods each have their limitations [126]. There is an urgent need to develop rapid, sensitive, and convenient novel detection methods. Gp50 is an engineered tail fibrin (ETFP) encoded by the lytic phage Abp9 and has been identified as a binding protein of *A*. *baumannii*. ETFP Gp50 serves as an ideal identification probe for rapidly screening *A*. *baumannii* strains in complex sample matrices, providing new technical means for the detection and control of drug-resistant *A*. *baumannii*, which is of great clinical and public health significance [127].

### 4.3. Route of Administration

#### 4.3.1. Topical Administration

Nebulization inhalation has been the primary method for delivering phage therapy to the respiratory tract via aerosolization (Table 3). By optimizing aerosol particle size (1–5 μm), phage deposition in alveoli can be increased to 42 ± 7%. Clinical cases have demonstrated that combining phage therapy with meropenem can reduce the time to sputum culture conversion to negative in CRAB pneumonia patients to 5 days, with phage concentrations in bronchoalveolar lavage fluid maintained at >10^6^ PFU/mL [128]. Nebulization of phage concentrate diluted with saline solution demonstrates good lytic efficacy against CRAB lung infections [102]. Using an ultrasonic humidifier to nebulize the phage stock solution into saline solution to produce a phage aerosol can also be used for environmental disinfection [117], effectively controlling nosocomial transmission of *A*. *baumannii*.

Local wound irrigation: A phage cocktail is irrigated into abscess cavities such as pancreatic pseudocysts, gallbladders, and the third abdominal cavity via a percutaneous catheter [129], enabling direct delivery of phages to the infected site, increasing local phage concentration, enhancing bactericidal efficacy, reducing phage impact on other systemic areas, and lowering potential adverse reactions.

Hydrogel formulation: Polyethylene glycol castor oil P407 and carbomer polymer C934P powder are mixed and dissolved in Milli-Q water, neutralized, and then combined with a phage suspension to prepare a thermosensitive hydrogel containing phages. On one hand, this helps maintain phage stability. For chronic wounds, the thermosensitive hydrogel carrier (polyethylene glycol castor oil P407/carbomer 934P) forms a three-dimensional network structure to achieve 72-h sustained release of phages, providing sufficient phage concentration at the infection site, extending antimicrobial duration, and enhancing antimicrobial efficacy; On the other hand, the phage gel can effectively target biofilms, providing a new treatment option for chronic wound infections [130].

#### 4.3.2. Systemic Administration

When local perfusion with phages yields unsatisfactory therapeutic effects, a phage cocktail can be administered to patients via intravenous injection [129]. Intravenous injection enables phages to rapidly enter the systemic circulation, promptly exerting therapeutic effects, particularly in cases of severe infection symptoms, where they can rapidly control the condition. Its serum half-life can reach 2.1 ± 0.3 h, and after PEG modification, it can evade complement system clearance, with concentrations increasing by 4.7 times in deep infection sites such as pancreatic abscesses [129]. Combined with local administration, this forms a treatment strategy combining systemic and local approaches.

### 4.4. Challenges and Limitations

In summary, phage therapy demonstrates considerable potential in managing *A. baumannii* infections, yet several challenges and limitations remain. In vitro, individual phages often exhibit a narrow host range—frequently restricted to specific subspecies or even strains—which limits their efficacy against diverse clinical isolates. Moreover, bacteria can rapidly develop resistance through mechanisms such as receptor modification, further constraining the utility of phage monotherapy. In vivo and clinical studies face additional complexities: high interpatient variability in immune responses and intricate pharmacokinetics complicate the design of individualized treatment regimens. Current clinical evidence relies heavily on case reports, with particularly scarce data from specific infection types such as systemic infections. Well-controlled, large-scale, randomized double-blind trials are still lacking to definitively establish the efficacy and safety of phage therapy. Future efforts should focus on standardizing phage production and application, conducting rigorously designed clinical trials with large cohorts, and elucidating the complex interactions between phages, the host, bacterial pathogens, and antibiotics. Based on this understanding of prophage-encoded defense systems [83], future precision therapy strategies urgently require incorporating “pre-treatment genomic assessment of target strains” into standard protocols. This involves systematically examining whether strains harbor lytic phages and their defense elements that could mediate treatment failure, enabling more accurate prognosis and phage selection. Simultaneously, the development of rapid diagnostic tools will be essential to guide phage selection, and improved localized delivery strategies should be pursued. Ultimately, these advances are critical to transition phage therapy from compassionate use and isolated cases toward standardized, evidence-based clinical practice.

## 5. Genetically Engineered Bacteriophages

### 5.1. Renovation Objectives and Key Engineering Strategies

In the context of the severe challenges posed by multidrug-resistant bacteria in antimicrobial therapy, phage engineering has emerged as a research hotspot in the field of infection control owing to its precise targeting and unique antimicrobial mechanisms (Table 4). However, natural phages have limitations, such as restricted host range, insufficient lytic efficiency, potential lysogenic risks, high immunogenicity, and functional specificity, making them unsuitable for direct application in clinical precision therapy [131]. The core objectives of engineered phage modification are twofold: first, to systematically overcome the aforementioned biological bottlenecks; second, to endow phages with precise targeting and spatiotemporal controllability, thereby constructing a “smart therapeutic system” capable of selectively eliminating pathogens and activating on demand, thus offering solutions that surpass traditional antibiotics [132].

Cutting-edge research has established a multidimensional engineering strategy framework to achieve these objectives. Expanding the host range is a key direction in phage engineering. Rational design based on receptor-binding protein (RBP) structure analysis, such as modifying the RBP ligand-binding domain or implementing heterologous tail fiber grafting, such as integrating the K1-5 phage tail fiber, can overcome host restrictions [133,134]. Additionally, the use of auxiliary phage-delivered lytic enzymes can bypass the adsorption step and directly degrade the cell wall. Enhancing lysis efficiency and overcoming drug resistance are core components in improving phage antimicrobial efficacy. This can be achieved by modifying chimeric endolysins to enhance phage penetration of biological membranes, introducing light-controlled or small-molecule-induced switches for precise lysis regulation, and expressing anti-CRISPR proteins to evade host defense systems [135]. To block lysogeny, CRISPR-mediated lysogeny gene knockout strategies can be employed, and capsid protein PEG modification can significantly reduce immunogenicity. In terms of functional enhancement, three main modification approaches exist: First, integrating fluorescent reporter genes (lux/luc) enables real-time monitoring of treatment; Second, vector-based modifications can deliver CRISPR-Cas9 targeting drug-resistant genes; and third, biofilm-degrading enzymes are expressed to synergistically eliminate persistent infection foci. Notably, a recent study revealed a novel bacterial defense system: Kiwa, a “supercomplex” embedded in the cell membrane that rapidly activates upon phage attachment to the bacterial surface, blocking viral DNA replication and transcription [136]. Given the characteristic that the DNA-binding domain of the Kiwa effector protein KwaB is susceptible to inhibition by phage Gam-like DNA mimic proteins, combined with strategies such as receptor-binding protein (RBP) grafting and CRISPR-Cas delivery already applied in the modification of *A. baumannii* phages [133], engineered phages expressing anti-KwaB inhibitory factors can be designed; simultaneously, leveraging the defensive redundancy between Kiwa and RecBCD (Gam-targeting competition), by modifying the phage genome to eliminate Gam homologs or introduce RecBCD inhibitory elements, the penetration efficiency of engineered phages into the membrane of *A. baumannii* can be synergistically enhanced.

**Table 4:** Comparison of engineering technologies.

Engineering Technology Type	Technical Principle	Advantages	Limitations	Key Differences
Classical Approaches (BRED method [137], Chemical Mutagenesis, Transduction)	Inducing mutagenesis via chemical agents or transferring exogenous genes via phages	Simple operation, minimal equipment requirements	High randomness, low efficiency, imprecise regulation; non-target mutations	Reliance on random mutagenesis; lacks directed modification capability; fundamentally distinct from modern precise editing
CRISPR-Cas Precision Editing [138]	Utilizing CRISPR-Cas systems for site-specific genomic modification (e.g., knockout, insertion, replacement)	High precision and efficiency for targeted gene modification; high editing efficiency and reproducibility	Off-target effects, potential for altered host tropism or enhanced virulence; requires comprehensive genomic data	Core of precise targeting; overcomes randomness of traditional methods; limited by understanding of gene function
HDR-Mediated Scarless Editing [139]	Removing exogenous marker sequences to ensure genomic integrity	Enhanced biosafety by avoiding risks from exogenous DNA	Complex operation, demanding experimental conditions	Focus on biosafety optimization; complements (e.g., CRISPR) rather than replaces existing editors
Modular Design with Standardized Biological Parts (BioBricks) [140]	Modularizing functional genes for rapid prototyping via standardized assembly	Streamlines processes and enhances reproducibility for accelerated translation; facilitates functional expansion	Potential module incompatibility issues; underdeveloped standardized parts libraries	Core of standardization & modularization; enhances efficiency & operability; facilitates technology transfer
Total Genome Synthesis and Rebooting [141]	De novo chemical synthesis or refactoring of genomes for customized functions	Overcoming natural genomic constraints for customizable functional modules; enables novel antimicrobial mechanisms	High synthesis costs, technically challenging; error-prone in long assembly	Shift from modifying existing to de novo design; overcomes functional limits of natural phages; highest technical barrier

### 5.2. Engineering Technology

Recombinant phages demonstrated significant efficacy in saving mice infected with lethal doses of *Staphylococcus aureus*. In a similar experimental system, lysis-deficient phages infecting *Pseudomonas aeruginosa* or *Escherichia coli* were more effective in protecting mice against lethal doses of these bacteria than wild-type phages, suggesting that lysis-deficient phages may have unique application potential in specific antimicrobial scenarios [142,143]. Temperate phages are generally considered unsuitable for traditional phage therapy because their unique adaptive effects on infected bacteria may trigger a series of unpredictable biological effects when applied in phage therapy, thereby limiting their application. Phage engineering can be used to address some of the adverse factors associated with these temperate phages. For example, Dedrick et al. reported a phage cocktail composed of three phages, two of which were BRED-modified temperate phages with forced lytic capabilities, which were recently successfully used to treat a cystic fibrosis patient with *Mycobacterium avium* complex infection following lung transplantation [137].

Currently, phage engineering research targeting multidrug-resistant *A*. *baumannii* is still in its developmental stage, with limited research findings. However, from the perspectives of theoretical analysis and application practices in other bacteria, this field demonstrates significant potential for future development [144]. Among these, the updating and iteration of technical methods are crucial for overcoming the limitations of natural phages and achieving precise antibacterial function. Traditional methods used for phage modification, such as chemical mutagenesis and transduction, have laid the foundation for related research but are limited in application due to their high randomness and low efficiency. In recent years, the integration of synthetic biology and genome-editing technologies has significantly accelerated the development of phage engineering. The CRISPR/Cas-based phage genome editing system, with its precise and efficient characteristics, has become the core technology for modification and has been validated in multiple studies [138]. Trace-free editing technology ensures the integrity of genetic information after phage genome modification, avoiding potential risks associated with the insertion of foreign sequences, thereby enhancing the biosafety of engineered phages [139]. The technology for the complete chemical synthesis and reconstruction of phage genomes enables the construction of functionally customized synthetic phages, overcoming the limitations of natural genomes and providing new pathways for expanding phage application scenarios [141]. The application of modular design and standardized biological components, such as BioBricks, promotes the standardization and reproducibility of phage modification processes, accelerating the translation of laboratory research findings into practical applications [140] and laying the foundation for the development of “plug-and-play” antimicrobial platforms.

### 5.3. Challenges and Safety

Although gene engineering technology has significantly enhanced the therapeutic potential of bacteriophages, engineered bacteriophages still face numerous challenges in their practical application. The translational pathway for engineered phage therapies requires a critical and balanced evaluation of their safety profile, particularly concerning systemic administration. This encompasses not only the risks inherent to genetic modification but also a detailed understanding of their interactions with the human host and a clear assessment of their overall therapeutic value.

#### 5.3.1. Immune Recognition and Altered Pharmacokinetics

Systemic administration, such as intravenous or intraperitoneal injection, introduces engineered phages directly into the circulatory system, where they are subject to immediate immune surveillance. A prevalent bioengineering strategy involves the functionalization of capsid proteins (e.g., through PEGylation) to circumvent immune detection and prolong plasma half-life. However, such surface modifications can themselves be immunogenic, potentially inducing the accelerated blood clearance phenomenon upon subsequent administrations, thereby abrogating the intended therapeutic advantage [145]. Furthermore, the interplay between phages and eukaryotic cells is multifaceted. Although certain phage types may exhibit inherent immunomodulatory properties, engineered virions with synthetically altered surfaces possess the potential to elicit unforeseen pro- or anti-inflammatory cascades, distinct from the responses triggered by their wild-type counterparts. For instance, wild-type phages have been documented to suppress NF-κB-mediated inflammatory responses in human cells [146].

#### 5.3.2. Engineered Vectors for Horizontal Gene Transfer

A paramount biosafety consideration is the capacity of engineered phages to act as efficient vectors for the horizontal gene transfer (HGT) of antibiotic resistance genes (ARGs). This risk manifests in several dimensions. Primarily, the molecular engineering workflow frequently relies on antibiotic resistance genes as selectable markers. The failure to ensure the complete excision of these marker genes from the final therapeutic construct establishes a direct conduit for the dissemination of ARGs into the host’s resident microbiota [147,148]. Second, and more critically, genetic manipulations designed to augment therapeutic efficacy can inadvertently increase the incidence of generalized transduction. Research has revealed that designing artificial pseudo-pac sequences highly similar to the P22 phage pac site and integrating them into the Salmonella genome significantly increases the probability of erroneous DNA packaging. This engineered modification elevates the transduction frequency in specific chromosomal regions by 10 to 100-fold, with transduction events concentrated near the pseudo-pac sites [149]. This finding underscores that even precise genetic enhancements can potentiate the innate ability of a phage to serve as a vehicle for resistance propagation. Finally, the inherent risk associated with lysogenic phages resident in the host microbiome is compounded by engineering interventions. Employing a temperate phage as a scaffold for genetic modification results in a stable, integrated prophage that serves as a reservoir for the engineered genome. Induction of this prophage can subsequently facilitate the dissemination not only of the engineered genetic elements but also of adjacent bacterial ARGs via specialized transduction [150].

In summary, while genetic engineering confers enhanced therapeutic capabilities, it simultaneously introduces and exacerbates distinct biosafety hazards. Consequently, a comprehensive risk assessment framework for engineered phage products must incorporate stringent screening for residual selection markers, empirical evaluation of HGT potential under physiologically relevant conditions, and detailed profiling of their immunopharmacological behavior (Table 5).

## 6. Conclusions

In summary, the primary ultimate goal of employing phages against CRAB is to achieve a paradigm shift from passive chemical-based bactericidal strategies to proactive bio-intelligent regulation. By integrating the ecological concept of “using bacteriophage to combat bacteria” with the engineering philosophy of “controlling virulence,” this approach aims to establish a sustainable and novel infection control system capable of countering the spread of drug-resistant bacteria.

## Figures and Tables

**Table 1:** Summary of the main classifications and characteristics of *Acinetobacter* phages.

Morphotype	Genus (Genomic Classification)	Representative Phages	Biological Characteristics and Genome	Host Range and Lytic Ability
Myovirus-like (Contractile tail with helical protein sheath; icosahedral capsid)	Phagecoctavirus	vB_AbaM-DLP_1,vB_AbaM-DLP_2 [57]	Elongated head; contractile tail with tail fibers; large burst size; short latency; stable over a broad pH range	Broad/unspecified range, high adaptability
	Obolenskvirus	Abp95 [58]	Broad spectrum (29%; 58/200); short latency; high burst size; rapid adsorption rate; depolymerase-containing; effective against diverse sequence types of CRAB	Broad activity across CRAB sequence types
		vB-AbaM-IME-AB2, WCHABP1, WCHABP12, BUCT628 [59], and HZY2308 [60]	Genomically classified as Obolenskvirus.	Varies by phage
	Unclassified	vB_AbaSi_W9 [61]	Broad host range; considered a potential therapeutic candidate despite lower lytic efficiency.	Broader than many myovirus-like phages
		vB_AbaM_AB3P2 [62]	Icosahedral head (70 nm diameter); tail 100 ± 10 nm long, 20 nm wide; potent lytic activity	Lytic only for *A. baumannii* strains AB3 & AB9; narrow host range
		vB_AbaM_ABMM1 (mild bacteriophage) [63]	Lysogenic (genome integration); rapid adsorption; large burst size; stable at neutral pH and temperature; effective in vitro and in vivo	Broad/unspecified range
		Phab24 [64], vB-GEC_Ab-M-G7 [65] and vB_AbaSi_W16 [66]	Morphologically and genetically similar to myovirus-like phages.	Varies by phage
Siphovirus-like (Long, non-contractile tail of unique morphology)	Friunavirus	Abp1 [67]	Standard icosahedral head; ~40–50 kb genome; encodes multiple biofilm penetration-associated genes	High specificity for host strain AB1; narrow host range
	Unclassified	DMU1 [68]	Long, striated, flexible tail, terminating in tail spikes and/or fibers	Infects only *A. baumannii* ATCC19606 & ATCC17978
		vB_AbaS_SA1 [69]	Latent period: 20 min; burst size: 250 PFU/cell; antibacterial efficacy against clinical MDR-AB	Targeting clinical multidrug-resistant (MDR) strains
Podovirus-like (Short, non-contractile tail)	Friunavirus	PD-6A3 [70]	Stable at 4–50 °C and pH 5–10; >90% adsorption within 5 min	Broad/unspecified range, high environmental adaptability
		MRABP9 [71]	Short latent period; large burst size; significant anti-biofilm activity; inhibits bacterial regrowth; high environmental stability	Targeting clinical MDR *A. baumannii* strains
	Unclassified	vB_AbaSi_W8 [61]	Lytic activity against clinical CRAB strains.	Narrower than vB_AbaSi_W9
		vB_AbaAut_ChT04 [72]	Latent period: 10 min; burst size: 280 PFU/cell; infects 52 of 150 clinical MDR-AB strains	Covers ~34.7% of clinical MDR strains

**Table 2:** Application Characteristics of Phage Therapy for *Acinetobacter baumannii* Infections in Various Disease.

Disease Type Treated	Study Model/Clinical Scenario	Key Phage(s)/Therapy	Core Efficacy & Characteristics	Limitations & Pending Issues
Respiratory Diseases	Mouse pneumonia model [20,21], Rat pneumonia model [22]; Clinical VAP [101], lung abscess	Single phage, Phage cocktail therapy; Phage + Antibiotic combination	Clears CRAB strains in animal models, improves survival, alleviates inflammation; Adjunct to antibiotics aids patient recovery	Narrow phage host range [100], prone to inducing resistance; Mechanisms of immune system impact unclear
Skin Wounds	Burn wounds [104], Diabetic foot ulcers [105] (rat model & clinical cases)	Phage cocktail [104]; Phage + Topical care products [106]/Low-dose antiseptic [107]	Clears drug-resistant bacteria from wounds and promotes healing in animal models; Combined care enhances antibacterial effect clinically	Requires prior pathogen strain identification; Few clinical cases for diabetic wounds, lack of blinded trials [108]
Bacteremia or Sepsis	Mouse MDR *A. baumannii* sepsis model [109]	vB_AbaM_3054, vB_AbaM_3090 (alone or combined) [109]	Efficiently clears pathogens and significantly improves infection symptoms in animal models	Lack of clinical application reports; Human safety and efficacy need validation; Complex interactions with the host immune system [110]
Urinary Tract Infections	Human urine model, Animal models	Phage cocktail [111]; Phage+ Trimethoprim/Sulfamethoxazole [112]	Inhibits biofilm formation in vitro [113], combination enhances antibacterial effect; No harm to normal flora [114]	High UTI recurrence rate requires optimized long-term regimen [115]; Insufficient clinical translation data
Hospital Transmission Control	ICU environment (CRAB contamination) [116]	Phage aerosol [117]	Reduces CRAB infection and resistance rates in ICU, aids environmental disinfection	Need to assess phage resistance development; Disinfection scope requires improvement [117]

**Table 3:** Administration Routes and Characteristics for *Acinetobacter baumannii* Phage Therapy.

Route Classification	Specific Method	Operational Procedure	Core Advantages	Application Scenarios
Topical Administration	Nebulized Inhalation [128]	1. Dilute phage stock with saline and administer via nebulizer [102]; 2. Use ultrasonic humidifier to aerosolize phage stock into saline, generating phage aerosol [117]	1. Delivers phages directly to the respiratory tract, lysing lung infection strains; 2. Enables rapid disinfection of large areas, controlling nosocomial transmission	1. Lung infections; 2. Disinfection of *A. baumannii* contamination in environments like ICUs
	Local Wound Perfusion	Percutaneous catheter perfusion of phage cocktail into abscess cavities (e.g., pancreatic pseudocyst, gallbladder, abdominal abscess) [129]	1. Acts directly on the infection site, increasing local phage concentration; 2. Reduces impact on other body areas, lowering adverse reaction risk	Diffuse drug-resistant *A. baumannii* infections
	Hydrogel Formulation	Mix polyethylene glycol castor oil P407, carbomer polymer C934P with phage suspension to prepare thermosensitive hydrogel [130]	1. Maintains phage stability, enables sustained release; 2. Targets biofilms, extends antimicrobial duration	Chronic wound infections
Systemic Administration	Intravenous Injection	Administer phage cocktail intravenously; can be combined with local administration [129]	1. Rapid entry into systemic circulation, timely control of severe infections; 2. Synergizes with local administration, enhances comprehensiveness	Severe infections unresponsive to local treatment; Systemic disseminated *A. baumannii* infection

**Table 5:** Risk-Benefit Analysis of Engineered Phage Therapeutics.

Aspect	Key Advantages	Key Risks & Disadvantages
Targeting & Efficacy	Expanded host range via RBP engineering. Enhanced biofilm penetration (e.g., via depolymerase expression). Ability to target antibiotic-tolerant persister cells.	Potential for off-target activity due to altered host range. Unpredictable inflammatory responses at infection sites.
Pharmacokinetics	Extended serum half-life through capsid PEGylation. Potential for targeted delivery to specific tissues.	Risk of Accelerated Blood Clearance (ABC) upon repeated dosing. Complex and costly pharmacokinetic profiling.
Genetic Stability & Safety	CRISPR-mediated removal of virulence/lysogeny genes. Expression of anti-CRISPR proteins to overcome bacterial defenses.	Genetic instability during manufacturing/passage. Amplified potential for Horizontal Gene Transfer (HGT) of ARGs. Potential for off-target effects from gene editing.
Regulatory & Manufacturing	“Designer” phages with tailored functionalities. Potential for standardized, off-the-shelf products.	Immense regulatory hurdles for live, replicating biologics. Complex, costly, and scaled manufacturing requirements. Lack of long-term environmental impact data.

## Data Availability

No data was used for the research described in the article.
